# Comparing pre- and post-COVID-19 chronic allergy prevalence in children using National Health and Nutrition Examination Survey in 2019 and 2021 

**Published:** 2026-04-02

**Authors:** Bomi Kim, Sunyeob Choi

**Affiliations:** 1 Department of Nursing, Honam University, Gwangju, and; 2 College of Nursing, Dongguk University WISE, Gyeongbuk, South Korea

**Keywords:** COVID-19, chronic allergic diseases, children, asthma, atopic dermatitis, allergic rhinitis, KNHANES

## Abstract

Objective: To investigate changes in demographic characteristics, parental smoking habits, and the prevalence of asthma, atopic dermatitis, and rhinitis among children in South Korea before and after the COVID-19 pandemic. Materials and methods: A retrospective analysis of national health survey data from 2019 and 2021 was conducted, including children aged 3 – 18 years. Factors such as gender, age, location, housing type, family size, income, body mass index, subjective health status, influenza vaccination, family structure, and parental smoking habits were analyzed. Results: No significant differences were found in most demographic characteristics and parental features between 2019 and 2021, except for influenza vaccination rates and mothers’ age at first childbirth. The influenza vaccination rate increased from 69.3% in 2019 to 77.8% in 2021, and the average maternal age at first birth increased from 28.46 years to 29.22 years. Asthma diagnoses showed no significant differences between the 2 years after adjusting for general and parent-related characteristics. For atopic dermatitis, significant differences in gender distribution were observed in 2021. Rhinitis diagnoses showed significant differences in age, area, and breastfeeding status between the two years. Conclusion: The COVID-19 pandemic may have influenced certain demographic characteristics, such as influenza vaccination rates and mothers’ age at first childbirth, but the prevalence of asthma, atopic dermatitis, and rhinitis among children remained largely unchanged between 2019 and 2021. This study underscores the importance of monitoring the impact of social changes on children’s health, particularly during significant events like the COVID-19 pandemic. Further research is required to understand the long-term effects of these changes on child health.

## Introduction 

Chronic allergic diseases, including asthma, atopic dermatitis, and allergic rhinitis, which occur during childhood, are gradually increasing due to increased triggering factors resulting from changes in living environments and weakened immune systems caused by a decrease in pediatric infectious diseases [1]. The prevalence of chronic allergic diseases in childhood is higher than in adulthood [2], and the prevalence rates of atopic dermatitis, asthma, and allergic rhinitis were 4.3, 3.2 [3], and 16.3% [4], respectively. Chronic allergic diseases are the most common chronic diseases in children [5, 6]. The World Allergy Organization defines children as the group with the highest burden related to allergic diseases [7]. Chronic diseases in children affect overall aspects of a child’s life, such as growth and development, emotional development, academics, and peer relationships [8, 9, 10, 11]. 

The Coronavirus Disease 2019 (COVID-19), an acute respiratory illness caused by the SARS-CoV-2 virus, which began in Wuhan, China, in December 2019, has become a global pandemic [12]. In Korea, since the first confirmed case of COVID-19 in January 2020, 250,000 people have been diagnosed with the virus, and 25,000 have died due to the virus [13]. Chronic allergic diseases such as asthma, atopic dermatitis, and allergic rhinitis have the characteristic of worsening due to respiratory-related viruses [14]. Also, mechanisms that trigger allergic reactions, such as cytokines, play a role in increasing the prevalence of COVID-19 in patients with allergic diseases [15], suggesting that COVID-19 may affect the prevalence of chronic allergic diseases in children. Recent findings indicate that pediatric patients with allergic rhinitis and aeroallergen sensitivity may exhibit milder COVID-19 symptoms, highlighting a complex interaction between allergic diseases and the virus. However, comprehensive data on the full impact of COVID-19 in children with chronic allergic conditions are still lacking [16]. 

In Korea, the prevalence of asthma and allergic rhinitis in adolescents significantly decreased in 2020 compared to 2019, but there was no change in the prevalence of atopic dermatitis [4]. However, a study targeting adults reported an increasing trend of asthma patients due to COVID-19, and a longer treatment period was required when diagnosed with COVID-19 [17]. In a study related to allergic rhinitis and COVID-19, 27% of COVID-19 patients experienced worsening rhinitis symptoms [18]. Although there was no relationship between the prevalence of atopic dermatitis and COVID-19 [19], some studies suggested that COVID-19 could exacerbate atopic dermatitis by triggering allergic reactions [15]. Therefore, there are varying results across studies regarding the relationship between COVID-19 and chronic allergic diseases. In this context, this study aims to clarify the difference in the prevalence of chronic allergic diseases in children before and after the outbreak of COVID-19 by using raw data from the Korea National Health and Nutrition Examination Survey in 2019 and 2021. By comparing the prevalence and risk factors of these diseases before and after the pandemic, the study seeks to provide a comprehensive understanding of the impact of COVID-19 on chronic allergic diseases in children and identify areas that may require further research or intervention. 

## Materials and methods 

### Data collection 

This study is a descriptive survey comparing raw data from the 8^th^ Korea National Health and Nutrition Examination Survey (KNHANES) [20] conducted between 2019 and 2021 by the Korea Centers for Disease Control and Prevention. KNHANES has been conducted annually since 1998, and it investigates health status, prevalence of chronic diseases, food intake and nutritional status, and health-related habits based on Article 16 of the National Health Promotional Act. The survey targets different age groups, including children (1 – 11 years old), adolescents (12 – 18 years old), and adults (19 years old and above), and investigates items specific to each group. The first case of COVID-19 in Korea was reported on January 20, 2020. Therefore, this study analyzes the raw data from 2019 and 2021 to compare the pre- and post-COVID-19 outbreak periods. 

### Study population 

The study population includes children aged 3 – 18 years old. Chronic allergic diseases are typically diagnosed during childhood and can have lifelong impacts [2]. This study focuses on children aged 3 years and older who are prone to chronic allergic diseases. The study’s inclusion criteria are children who have been diagnosed with asthma, atopic dermatitis, or allergic rhinitis by a doctor or are currently suffering from these diseases. To clarify the relationship between the prevalence of children’s chronic allergic diseases and parental factors, this study also includes children whose parents have completed the survey. A total of 791 participants from 2019 and 562 participants from 2021 were included in the study. 

### Variables 

The study includes variables related to the prevalence of chronic allergic diseases, such as children’s characteristics, family characteristics, and parental characteristics, as collected by the KNHANES. Children’s characteristics include gender, age (pre-school, school-age, adolescence), body mass index (BMI) (< 5^th^ percentile, 5 – 85^th^ percentile, 85 – 95^th^ percentile, ≥ 95^th^ percentile), self-perceived health status (good, average, poor), and influenza vaccination status. Family characteristics include residential area (city, province), type of housing (general housing, apartment), family income level (low, lower-middle, middle, upper-middle, high), and family generation structure (2 generations, 3 generations or more). Parental factors include age at childbirth and breastfeeding status. Outcome variables include the current status of doctor’s diagnosis. 

### Ethical considerations 

This study was conducted using publicly available data from the Korean National Health and Nutrition Examination Survey (KNHANES) [20]. The KNHANES is conducted annually and is approved by the Institutional Review Board of the Korea Centers for Disease Control and Prevention (Approval No. 2018-01-03-3C-A). The data used in this study was downloaded from the KNHANES website after following the appropriate procedures, and all provided data was de-identified and cannot be used to identify individual subjects. 

### Data analysis 

To evaluate the general population characteristics of 2019 and 2021, we conducted linear regression analysis using complex sampling, and applied the χ^2^-test with Rao-Scott correction to the population data with weighted values. All results were presented as weighted values using the weights recommended by KNHANES. The χ^2^-test was used to compare the prevalence and diagnosis of chronic allergic diseases by year based on individual characteristics, family characteristics, and parental characteristics. 

Two sets of adjusted analyses were performed. Adjusted1 included only the general characteristics of the child as confounding variables, while Adjusted2 included the general characteristics of the child, as well as parental smoking-related variables, mother’s age at first childbirth, and breastfeeding status. The analysis was further adjusted for potential confounders, such as age, gender, and other relevant factors. 

All statistical analyses were conducted using SPSS 28.0 version. A p-value of less than 0.05 was considered statistically significant. The results of the data analysis were used to identify any differences in the prevalence and risk factors of chronic allergic diseases before and after the COVID-19 outbreak. Additionally, the findings were compared and contrasted with existing literature to provide context and further understanding of the impact of COVID-19 on childhood chronic allergic diseases. 

## Results 

### Comparison of general and parent-related characteristics of children in 2019 and 2021 

In this study, we compared the distribution of various factors between 2019 and 2021. These factors include gender, age, location (urban/rural), type of housing, family size, income, BMI, subjective health status, influenza vaccination, family structure, parental smoking habits, and maternal breastfeeding. There were no significant differences between the 2 years for most factors, except for the influenza vaccination rate (p = 0.005) and maternal age at first birth (p = 0.046). The influenza vaccination rate increased from 69.3% in 2019 to 77.8% in 2021, and the average maternal age at first birth increased from 28.46 years to 29.22 years (Table 1). 

### Comparison of general and parent-related characteristics of children diagnosed with asthma in 2019 and 2021 

We compared the general and parent-related characteristics of children diagnosed with asthma in 2019 and 2021. After adjusting for general and parent-related characteristics, there were no significant differences found in asthma diagnoses between the 2 years. Gender, age, residence, housing type, household composition, household income, BMI, subjective health status, and influenza vaccination status were similar in both years. Parental smoking habits and other parent-related variables also showed no significant differences between the 2 years (Table 2). 

### Comparison of general and parent-related characteristics of children diagnosed with atopic dermatitis in 2019 and 2021 

Gender distribution was not significantly different in 2019 data, but was in 2021 data (p = 0.014). Age distribution, type of housing, and parental variables showed no significant differences in both years. However, family size showed a significant difference in 2021 (p = 0.019), which was not observed in 2019. Significant differences were observed in income levels between the 2 years (p < 0.001 in 2021) and in BMI percentiles (p = 0.021 in 2019 and p = 0.418 in 2021). Subjective health status also showed significant differences in both years (p = 0.023 in 2019 and p < 0.001 in 2021). 

In the 2019 data, there was no significant difference in gender distribution, but in the 2021 data, there was a significant difference (p = 0.014), indicating a change in gender distribution in 2021. Age distribution, type of housing, family size, and parental variables showed no significant differences in both years. Income levels showed significant differences between the 2 years (p < 0.001). BMI percentiles showed a significant difference in 2019 (p = 0.021), indicating a decrease in 2021, but no significant difference in 2021 (p = 0.418). Subjective health status also showed significant differences in both years (p = 0.023 in 2019 and p < 0.001 in 2021), with a decline in subjective health status observed in 2019 and an improvement in 2021 (Table 3). 

### Comparison of general and parent-related characteristics of children diagnosed with rhinitis in 2019 and 2021 

In 2019, several general characteristics showed statistically significant differences in rhinitis diagnosis, including gender (p < 0.001), residential area (p = 0.040), and influenza vaccination status (p = 0.035). Specifically, male children had a significantly higher rate of diagnosis in 2019. However, in 2021, these variables were distributed similarly between the two groups, showing no statistical significance. Instead, the 2021 data revealed new significant factors: age (p = 0.012) and family size (p < 0.001). The average age was higher in the diagnosed group in 2021, and the diagnosis rate was notably lower in the 3 – 6 years age group (p = 0.026). Regarding parental characteristics, breastfeeding history was a significant factor only in 2019 (p = 0.005), whereas mother’s age at first childbirth showed a significant difference only in 2021 (p = 0.007). Other variables, such as housing type, household income, BMI, and parental smoking status, showed no statistically significant differences in either year (Table 4). 

## Discussion 

This study compared the demographic characteristics and parental smoking habits of children in 2019 and 2021. The results found little difference between the years in most demographic characteristics and parental features, but there were significant differences in influenza vaccination and mothers’ age at first childbirth. Influenza vaccination rates were relatively higher in 2021 compared to 2019, which has been observed in previous studies during the COVID-19 pandemic [21]. This change may be interpreted as an increased interest in influenza vaccination as awareness of vaccines due to COVID-19 increased. There is limited research on the effect of the pandemic on mothers’ age at first childbirth, but it is believed to be associated with a temporary decrease in birth rates. Social and economic changes during the COVID-19 pandemic may have influenced decisions related to childbirth [22]. Unconsidered factors may have contributed to the observed changes in the age of first childbirth in this study. 

This study aimed to compare the demographic characteristics and parental smoking habits of children diagnosed with asthma in 2019 and 2021, reflecting the social changes before and after the COVID-19 pandemic in South Korea. The results of this study did not reveal any significant differences in demographic characteristics or parental smoking habits between the 2 years. Similar findings have been reported in previous studies [23, 24]. Considering the social changes due to the COVID-19 pandemic, several interpretations can be drawn from these findings. 

First, the main factors influencing asthma diagnosis may not have changed significantly between 2019 and 2021 despite the pandemic. However, it is important to consider that the prevalence of atopic dermatitis diagnoses tends to decrease as children age. Studies have demonstrated that younger children exhibit higher rates of atopic dermatitis diagnosis compared to older children and adolescents. For instance, the prevalence rates of atopic dermatitis and wheeze decrease with age, while the prevalence of allergic rhinitis tends to increase [25, 26]. This trend suggests that age is a significant factor in the diagnosis of atopic dermatitis, highlighting the importance of targeted prevention and management strategies that address the needs of younger children who are more likely to be diagnosed with the condition. Furthermore, existing prevention and management strategies may still be effective during this period, particularly among younger children. This aligns with findings from previous studies indicating that early intervention can mitigate the severity and progression of atopic dermatitis [27, 28]. 

Second, other factors influenced by social changes brought about by the pandemic may have affected asthma diagnosis. Increased indoor living, changes in air pollution levels, and alterations in lifestyle habits are among the factors that have been identified. For example, the pandemic led to significant lifestyle changes, including more time spent indoors and reduced physical activity, which can exacerbate symptoms of asthma and atopic dermatitis [29, 30]. Additionally, changes in environmental factors, such as air pollution, during the pandemic have had a notable impact on the prevalence and severity of atopic dermatitis in children [31]. 

In both 2019 and 2021, our study found that the prevalence of atopic dermatitis diagnosis was highest among children aged 7 – 11 years. However, it is essential to recognize that while some children may experience a decrease in symptoms or complete resolution of atopic dermatitis as they grow older, others may continue to experience symptoms into adolescence and adulthood [32]. The persistence or resolution of atopic dermatitis may be influenced by various factors, including genetic predisposition, environmental exposures, and the individual’s immune response [27]. 

The association between gender and atopic dermatitis diagnosis was not significant in 2019 but became significant in 2021. This finding aligns with a recent study that reported higher prevalence rates of atopic dermatitis among boys compared to girls, with significant differences observed particularly in younger age groups, such as infants and toddlers [33]. These findings suggest that boys may be more susceptible to developing atopic dermatitis in early childhood due to various factors, including differences in immune system development and environmental exposures. Regarding the living environment, the type of housing did not reach statistical significance in either year. This result diverges from some previous studies [34] that have strongly linked specific housing types or increased urbanization to the prevalence of atopic dermatitis. Children living in urban areas, particularly in high-density housing, have higher exposure to indoor allergens and pollutants, which can exacerbate or trigger atopic dermatitis symptoms. Furthermore, the number of people in the family was not significantly associated with atopic dermatitis diagnosis in 2019 but showed a significant association in 2021. This finding is consistent with a study by Pelucchi et al. [35], which reported that a larger family size may have a protective effect against atopic dermatitis, possibly due to increased exposure to diverse microorganisms that can modulate the immune system. 

Regarding variables related to rhinitis, the significant difference in breastfeeding status between the 2 years could be related to changes in healthcare practices or parental behavior during the COVID-19 pandemic. A study conducted by La Verde et al. [36] found that the pandemic may have affected the availability of healthcare services, including breastfeeding support, as well as the accessibility of information on breastfeeding, leading to altered breastfeeding practices. The authors reported that breastfeeding initiation rates decreased during the pandemic, possibly due to the increased stress and anxiety experienced by mothers. The COVID-19 pandemic might have also contributed to the observed differences in age, area, and number of family members among children diagnosed with rhinitis. Changes in environmental factors, increased time spent indoors, and altered hygiene practices during the pandemic could have affected the development of rhinitis in children [37]. According to a study by Li et al. [38], increased exposure to indoor allergens and irritants during the pandemic could have contributed to a higher incidence of allergic diseases, including rhinitis, in children. Additionally, the study found that families living in crowded conditions were more likely to have children diagnosed with rhinitis. 

This study provides a comprehensive comparison of the general and parent-related characteristics of children diagnosed with asthma, atopic dermatitis, and rhinitis in 2019 and 2021, taking into account the potential impact of the COVID-19 pandemic. The research design allows for a thorough analysis of various factors, including demographic characteristics, parental smoking habits, and environmental factors, that might influence the prevalence and diagnosis of these diseases. By linking the results to previous research and offering plausible explanations for the observed trends, this study contributes to the existing knowledge on the factors affecting childhood asthma, atopic dermatitis, and rhinitis in South Korea. 

The main limitations of this study are as follows. First, the study relies on self-reported measures instead of objective indicators for diagnosing and determining the prevalence of chronic allergic diseases. This can lead to the possibility of underreporting or overreporting, reducing the reliability of the findings. Second, the study is cross-sectional, which makes it difficult to establish causal relationships. In particular, it is impossible to completely rule out the influence of selection bias and confounding variables, and there might be other uncontrolled confounding factors that could affect the results. Third, the study aimed to examine the relationship between COVID-19-related characteristics, such as vaccination status, handwashing, and mask-wearing, and chronic allergic diseases. However, the original dataset did not include these variables, limiting the analysis. This makes it difficult to accurately estimate the impact of the COVID-19 pandemic on chronic allergic diseases. Last, the sample size is relatively small, and the study focuses on South Korea, which may limit the generalizability of the findings to other countries or populations with different cultural, socioeconomic, or environmental contexts. This limitation restricts the applicability of the study’s results. 

## Conclusion 

In conclusion, this study provides a comprehensive comparison of the general and parent-related characteristics of children diagnosed with asthma, atopic dermatitis, and rhinitis in South Korea in 2019 and 2021, considering the potential impact of the COVID-19 pandemic. The results revealed several significant differences between the 2 years in certain aspects, while other factors remained stable. This highlights the complex interplay of demographic, parental, environmental, and social factors that influence the prevalence and diagnosis of these diseases in children. Furthermore, the study emphasizes the importance of considering the broader social and environmental context, including the potential influence of the COVID-19 pandemic, when developing and implementing prevention and management strategies for childhood asthma, atopic dermatitis, and rhinitis. As our understanding of these diseases continues to evolve, it is crucial to adapt and refine prevention and management approaches to address the changing landscape and emerging challenges. Overall, this study contributes valuable insights to the existing knowledge on factors affecting childhood asthma, atopic dermatitis, and allergic rhinitis in South Korea and underscores the need for continued research and collaboration among researchers, healthcare professionals, policymakers, and other stakeholders to improve the health and well-being of children affected by these conditions. 

## Authors’ contributions 

Bomi Kim: Contributed to conceptualization, data curation, formal analysis, and writing of the original draft. 

Sunyeob Choi: Contributed to supervision, methodology, and review and editing of the manuscript. 

## Funding 

This research received no external funding. 

## Conflict of interest 

There is no conflict of interest. 

**Table 4.** Comparison of general and parent-related characteristics of children diagnosed with rhinitis diagnosis in 2019 and 2021

		**Rhinitis diagnosis**									
**Year**		**2019 (n = 791)**			**2021 (n = 562)**						
		**M ± SD, n (%)**			**M ± SD, n (%)**						
		**Yes**	**No**	**F (p)**	**Yes**	**No**	**F (p)**	**OR**	**95% CI**		**p**
General characteristics											
Gender	Male	130 (16.4)	278 (35.1)	10.120 (< 0.001)	80 (14.2)	198 (35.2)	0.650 (0.422)	0.842	0.547	1.297	0.434
	Female	84 (10.6)	299 (37.8)		72 (12.8)	212 (37.7)		1.210	0.728	2.009	0.461
Age	M ± SD	9.29 ± 3.70	9.52 ± 4.28	–1.350 (0.180)	10.21 ± 3.67	9.42 ± 4.10	2.556 (0.012)	0.986	0.679	1.432	0.941
	3 – 6	54 (6.8)	173 (21.9)	1.707 (0.184)	26 (4.6)	119 (21.2)	3.723 (0.026)	0.583	0.306	1.111	0.101
	7 – 11	94 (11.9)	214 (27.1)		71 (12.6)	148 (26.3)		0.960	0.548	1.683	0.886
	12 – 17	66 (8.3)	190 (24.0)		55 (9.8)	143 (25.4)		1.344	0.782	2.308	0.283
Residential area	Urban	185 (23.4)	484 (61.2)	4.325 (0.040)	135 (24.0)	336 (59.58)	0.826 (0.365)	0.954	0.647	1.408	0.813
	Rural	29 (3.7)	93 (11.8)		17 (3.0)	74 (13.2)		1.319	0.410	4.242	0.641
Type of housing	General housing	40 (5.1)	129 (16.3)	2.123 (0.148)	27 (4.8)	113 (20.1)	2.465 (0.119)	0.967	0.456	2.052	0.930
	Apartment	174 (22.0)	448 (56.6)		125 (22.2)	297 (52.8)		0.993	0.653	1.511	0.975
Number in family		4.11 ± 0.68	4.20 ± 0.76	–1.125 (0.263)	4.01 ± 0.71	4.18 ± 0.76	–3.423 (< 0.001)	0.963	0.667	1.391	0.841
Income	Low	2 (0.3)	12 (1.5)	0.649 (0.614)	4 (0.7)	2 (0.4)	0.928 (0.442)	12.588	0.797	198.944	0.072
	Lower middle	30 (3.8)	99 (12.5)		14 (2.5)	44 (7.8)		0.872	0.278	2.732	0.814
	Middle	60 (7.6)	149 (18.8)		43 (7.7)	130 (23.1)		0.777	0.412	1.466	0.435
	Upper middle	70 (8.8)	193 (24.4)		55 (9.8)	132 (23.5)		1.135	0.668	1.928	0.639
	High	52 (6.6)	124 (15.7)		36 (6.4)	102 (18.1)		0.948	0.477	1.885	0.880
Body mass index	< 5^th^ percentile	15 (1.9)	54 (6.8)	0.769 (0.507)	11 (2.0)	23 (4.1)	1.217 (0.303)	2.739	0.851	8.817	0.091
	5 – 85^th^ percentile	146 (18.5)	417 (52.8)		101 (18.2)	281 (50.5)		0.943	0.612	1.453	0.789
	85 – 95^th^ percentile	24 (3.0)	49 (6.2)		21 (3.8)	43 (7.7)		1.170	0.544	2.518	0.686
	≥ 95^th^ percentile	29 (3.7)	56 (7.1)		19 (3.4)	57 (10.3)		0.675	0.278	1.636	0.382
Subjective health status	Good	149 (18.8)	437 (55.2)	1.635 (0.199)	113 (20.1)	316 (56.2)	0.048 (0.949)	1.041	0.679	1.598	0.852
	Fair	57 (7.2)	125 (15.8)		33 (5.9)	81 (14.4)		0.917	0.502	1.678	0.779
	Poor	8 (1.0)	15 (1.9)		6 (1.1)	13 (2.3)		0.590	0.15	2.322	0.449
Influenza vaccination	Yes	163 (20.6)	385 (48.7)	4.553 (0.035)	129 (23.0)	308 (54.8)	3.264 (0.074)	0.927	0.606	1.418	0.727
	No	51 (6.4)	192 (24.3)		23 (4.1)	102 (18.1)		0.961	0.46	2.006	0.915
Household composition	2 generations	202 (25.5)	539 (68.1)	0.001 (0.978)	142 (25.3)	393 (69.9)	0.892 (0.347)	0.958	0.664	1.383	0.818
	3 generations or more	12 (1.5)	38 (4.8)		10 (1.8)	17 (3.0)		1.765	0.371	8.403	0.474
Parental characteristics											
Father’s smoking history	< 5 packs	4 (0.5)	5 (0.6)	1.524 (0.220)	7 (1.2)	8 (1.4)	2.546 (0.083)	1.031	0.094	11.305	0.980
	≥ 5 packs	154 (19.5)	448 (56.6)		121 (21.5)	304 (54.1)		1.177	0.798	1.735	0.410
	Never smoked	56 (7.1)	124 (15.7)		24 (4.3)	98 (17.4)		0.509	0.246	1.054	0.069
Father’s current smoking status	Daily	71 (9.0)	176 (22.3)	1.776 (0.152)	54 (9.6)	123 (21.9)	0.783 (0.494)	1.005	0.578	1.748	0.985
	Occasionally	11 (1.4)	27 (3.4)		6 (1.1)	23 (4.1)		0.710	0.132	3.823	0.689
	Former smoker	76 (9.6)	250 (31.6)		68 (12.1)	166 (29.5)		1.440	0.872	2.377	0.153
	Never smoked	56 (7.1)	124 (15.7)		24 (4.3)	98 (17.4)		0.509	0.246	1.054	0.069
Mother’s smoking history	< 5 packs	6 (0.8)	14 (1.8)	0.785 (0.454)	2 (0.4)	17 (3.0)	1.225 (0.296)	0.249	0.016	3.810	0.317
	≥ 5 packs	27 (3.4)	57 (7.2)		17 (3.0)	40 (7.1)		0.662	0.277	1.582	0.352
	Never smoked	181 (22.9)	506 (64.0)		133 (23.7)	353 (62.8)		1.090	0.736	1.616	0.665
Mother’s current smoking status	Daily	9 (1.1)	5 (0.6)	1.909 (0.139)	3 (0.5)	14 (2.5)	0.916 (0.433)	0.104	0.013	0.845	0.034
	Occasionally	5 (0.6)	11 (1.4)		0 (0.0)	6 (1.1)					
	Former smoker	19 (2.4)	55 (7.0)		16 (2.8)	37 (6.6)		0.942	0.347	2.555	0.906
	Never smoked	181 (22.9)	506 (64.0)		133 (23.7)	353 (62.8)		1.090	0.736	1.616	0.665
Mother’s age at first childbirth		28.57 ± 3.65	28.42 ± 3.90	0.437 (0.663)	30.0 ± 3.16	28.93 ± 3.88	2.764 (0.007)	0.942	0.651	1.364	0.751
Mother’s breastfeeding history	Yes	182 (23.0)	529 (66.9)	8.286 (0.005)	135 (24.0)	369 (65.7)	0.847 (0.360)	1.060	0.722	1.554	0.766
	No	32 (4.0)	48 (6.1)		17 (3.0)	41 (7.3)		0.591	0.219	1.596	0.298
Crude								0.986	0.678	1.431	0.941
Adjusted1 (includes general characteristics)								0.918	0.634	1.329	0.649
Adjusted2 (includes general and parental characteristics)								0.862	0.596	1.248	0.431

**Table 1.** Comparison of general and parent-related characteristics of children in 2019 and 2021.

**Variable**	**Category**	**Year**		**χ** *^2^* ** or t**	**p-value**
		**M ± SD, n (%)**			
		**2019 (n = 791)**	**2021 (n = 562)**		
Gender	Male	408 (51.6)	278 (49.5)	0.717	0.398
	Female	383 (48.4)	284 (50.5)		
Age	Mean ± SD	9.46 ± 4.134	9.63 ± 4.000	0.157	0.875
	3 – 6	277 (28.7)	145 (25.8)	0.240	0.780
	7 – 11	308 (38.9)	219 (39.0)		
	12 – 17	256 (32.4)	198 (35.2)		
Rural/urban	Urban	669 (84.6)	471 (83.8)	0.007	0.932
	Rural	122 (15.4)	91 (16.2)		
Type of housing	General housing	169 (21.4)	140 (24.9)	0.007	0.935
	Apartment	622 (78.6)	422 (75.1)		
Number in family		4.17 ± 0.739	4.14 ± 0.750	–1.012	0.313
Income	Low	14 (1.8)	6 (1.1)	0.978	0.414
	Lower middle	129 (16.3)	58 (10.3)		
	Middle	209 (26.4)	173 (30.8)		
	Upper middle	263 (33.2)	187 (33.3)		
	High	176 (22.3)	138 (24.6)		
Body mass index	< 5^th^ percentile	69 (8.7)	34 (6.0)	1.308	0.271
	5 – 85^th^ percentile	563 (71.2)	382 (68.0)		
	85 – 95^th^ percentile	73 (9.2)	64 (11.4)		
	≥ 95^th^ percentile	85 (10.7)	76 (13.5)		
Subjective health status	Good	586 (74.1)	429 (76.3)	0.535	0.580
	Fair	182 (23.0)	114 (20.3)		
	Poor	23 (2.9)	19 (3.4)		
Influenza vaccination	Yes	548 (69.3)	437 (77.8)	8.040	0.005
	No	243 (30.7)	125 (22.2)		
Household composition	2 generations	741 (93.7)	535 (95.2)	1.919	0.167
	3 generations or more	50 (6.3)	27 (4.8)		
Father					
Lifetime smoking amount	< 5 packs	9 (1.1)	15 (2.7)	1.066	0.344
	≥ 5 packs	602 (76.1)	425 (75.6)		
	Never smoked	180 (22.8)	122 (21.7)		
Current smoking status	Daily	247 (31.2)	177 (31.5)	0.215	0.885
	Occasionally	38 (4.8)	29 (5.2)		
	Former smoker	326 (41.2)	234 (41.6)		
	Never smoked	180 (22.8)	122 (21.7)		
Mother					
Lifetime smoking amount	< 5 packs	20 (2.5)	19 (3.4)	0.544	0.581
	≥ 5 packs	84 (10.6)	57 (10.1)		
	Never smoked	687 (86.9)	486 (86.5)		
Current smoking status	Daily	14 (1.8)	17 (3.0)	0.289	0.807
	Occasionally	16 (2.0)	6 (1.1)		
	Former smoker	74 (9.4)	53 (9.4)		
	Never smoked	687 (86.9)	486 (86.5)		
Mother’s age at first childbirth	Mean (SD)	28.46 ± 0.136	29.22 ± 3.731	2.002	0.046
Mother’s breastfeeding status	Yes	711 (89.9)	504 (89.7)		
	No	80 (10.1)	58 (10.3)	0.240	0.625

**Table 2.** Comparison of general and parent-related characteristics of children diagnosed with asthma in 2019 and 2021.

		**Asthma diagnosis**									
**Year**		**2019 (n = 791)**			**2021 (n = 562)**						
		**M ± SD, n (%)**			**M ± SD, n (%)**						
		**Yes**	**No**	**χ** *^2^* ** (p)**	**Yes**	**No**	**χ** *^2^* ** (p)**	**OR**	**95% CI**		**p**
General characteristics											
Gender	Male	19 (2.4)	389 (49.2)	7.183 (0.008)	7 (1.2)	271 (48.2)	5.368 (0.022)	0.522	0.030	0.089	0.211
	Female	3 (0.4)	380 (48.0)		3 (0.5)	281 (50.0)		0.577	0.109	3.039	0.515
Age	M ± SD	10 ± 4.220	9.44 ± 4.133	0.167 (0.868)	10.30 ± 3.302	9.62 ± 4.013	0.828 (0.408)	0.518	0.212	1.266	0.148
	3 – 6	4 (0.5)	223 (28.2)	0.317 (0.716)	1 (0.2)	144 (25.6)	0.712 (0.478)	0.203	0.021	1.933	0.165
	7 – 11	11 (1.4)	297 (37.5)		6 (1.1)	213 (37.9)		0.550	0.198	1.525	0.249
	12 – 17	7 (0.9)	249 (31.5)		3 (0.5)	195 (34.7)		0.646	0.118	3.529	0.612
Residential area	Urban	19 (2.4)	650 (82.2)	1.308 (0.255)	7 (1.2)	464 (82.6)	0.547 (0.461)	0.442	0.159	1.225	0.116
	Rural	3 (0.4)	119 (15.0)		3 (0.5)	88 (15.7)		1.520	0.296	7.811	0.614
Type of housing	General housing	4 (0.5)	165 (20.9)	0.447 (0.505)	3 (0.5)	137 (24.4)	0.033 (0.856)	0.789	0.178	3.510	0.755
	Apartment	18 (2.3)	604 (76.4)		7 (1.2)	415 (73.8)		0.462	0.155	1.373	0.164
Number in family		3.91 ± 0.684	4.18 ± 0.739	–1.735 (0.085)	4 ± 0.667	4.14 ± 0.752	–0.954 (0.341)	0.493	0.205	1.185	0.113
Income	Low	0 (0.0)	14 (1.8)	0.413 (0.778)	0 (0.0)	6 (1.1)	0.744 (0.454)				
	Lower middle	5 (0.6)	124 (15.7)		2 (0.4)	56 (10.0)		0.632	0.107	3.714	0.610
	Middle	2 (0.3)	207 (26.2)		4 (0.7)	169 (30.1)		1.437	0.181	11.387	0.731
	Upper middle	11 (1.4)	252 (31.69)		1 (0.2)	186 (33.1)		0.110	0.014	0.869	0.036
	High	4 (0.5)	172 (21.7)		3 (0.5)	135 (24.0)		0.558	0.097	3.227	0.514
Body mass index	< 5^th^ percentile	1 (0.1)	68 (8.6)	0.150 (0.918)	0 (0.0)	34 (6.1)	0.698 (0.539)				
	5 – 85^th^ percentile	17 (2.2)	546 (69.1)		9 (1.6)	373 (67.1)		0.642	0.236	1.747	0.384
	85 – 95^th^ percentile	2 (0.3)	71 (9.0)		1 (0.2)	63 (11.3)		0.509	0.038	6.775	0.608
	≥ 95^th^ percentile	2 (0.3)	83 (10.5)		0 (0.0)	76 (13.7)					
Subjective health status	Good	15 (1.9)	571 (72.2)	0.504 (0.598)	7 (1.2)	422 (75.1)	6.455 (0.004)	0.547	0.179	1.674	0.289
	Fair	7 (0.9)	175 (22.1)		1 (0.2)	113 (20.1)		0.068	0.008	0.573	0.014
	Poor	0 (0.0)	23 (2.9)		2 (0.4)	17 (3.0)					
Influenza vaccination	Yes	15 (1.9)	533 (67.4)	0.806 (0.371)	7 (1.2)	430 (76.5)	0.005 (0.945)	0.443	0.147	1.330	0.146
	No	7 (0.9)	234 (29.8)		3 (0.5)	122 (21.7)		0.761	0.171	3.391	0.719
Household composition	2 generations	21 (2.7)	720 (91.0)	0.421 (0.518)	10 (1.8)	525 (93.4)	0.218 (0.642)	0.524	0.212	1.292	0.160
	3 generations or more	1 (0.1)	49 (6.2)		0 (0.0)	27 (4.8)					
Parental characteristics											
Father’s smoking history	< 5 packs	0 (0.0)	9 (1.1)	0.147 (0.769)	0 (0.0)	15 (2.7)	0.808 (0.443)				
	≥ 5 packs	11 (1.4)	591 (74.7)		8 (1.4)	417 (74.2)		0.868	0.314	2.400	0.785
	Never smoked	11 (1.4)	169 (21.4)		2 (0.4)	120 (21.4)		0.275	0.042	1.786	0.175
Father’s current smoking status	Daily	1 (0.1)	246 (31.1)	0.169 (0.886)	3 (0.5)	174 (31.0)	0.091 (0.945)	2.056	0.206	20.478	0.538
	Occasionally	2 (0.3)	36 (4.6)		3 (0.2)	28 (5.0)		0.579	0.039	8.527	0.690
	Former smoker	8 (1.0)	318 (40.2)		4 (0.7)	230 (40.9)		0.746	0.225	2.476	0.631
	Never smoked	11 (1.4)	169 (21.4)		2 (0.4)	120 (21.4)		0.275	0.042	1.786	0.175
Mother’s smoking history	< 5 packs	1 (0.1)	19 (2.4)	0.120 (0.787)	1 (0.2)	18 (3.2)	1.968 (0.143)	3.215	0.168	61.59	0.437
	≥ 5 packs	2 (0.3)	82 (10.4)		0 (0.0)	57 (10.1)		< 0.001	5.29E-12	8.06E-11	**< 0.001**
	Never smoked	19 (2.4)	668 (84.5)		9 (1.6)	477 (84.9)		0.55	0.21	1.442	0.223
Mother’s current smoking status	Daily	0 (0)	14 (1.8)	0.201 (0.866)	0 (0.0)	17 (3.0)	0.135 (0.931)				
	Occasionally	0 (0)	16 (2.0)		0 (0.0)	6 (1.1)					
	Former smoker	3 (0.4)	71 (9.0)		1 (0.2)	52 (9.3)		0.297	0.027	3.254	0.319
	Never smoked	19 (2.4)	668 (84.5)		9 (1.6)	477 (84.9)		0.55	0.21	1.1442	0.223
Mother’s age at first childbirth		27.82 (3.78)	28.56 (3.90)	–0.165 (0.869)	30.10 (2.73)	29.20 (3.75)	1.025 (0.308)	0.52	0.213	1.266	0.149
Mother’s breastfeeding history	Yes	22 (2.8)	689 (87.1)	2.045 (0.155)	9 (1.6)	495 (88.1)	0.020 (0.889)	0.373	0.165	0.845	0.018
	No	0 (0)	80 (10.1)		1 (0.2)	57 (10.1)					
Crude								0.519	0.213	1.265	0.148
Adjusted1 (includes general characteristics)								0.471	0.199	1.117	0.087
Adjusted2 (includes general and parental characteristics)								0.541	0.231	1.265	0.541

**Table 3.** Comparison of general and parent-related characteristics of children diagnosed with atopic dermatitis diagnosis in 2019 and 2021.

		**Atopic dermatitis diagnosis**									
**Year**		**2019 (n = 791)**			**2021 (n = 562)**						
		**M ± SD, n (%)**			**M ± SD, n (%)**						
		**Yes**	**No**	**χ** ^2^ ** (p)**	**Yes**	**No**	**χ** ^2^ ** (p)**	**OR**	**95% CI**		**p**
General characteristics											
Gender	Male	56 (7.1)	352 (44.5)	0.005 (00.945)	45 (8.0)	233 (41.5)	6.305 (0.014)	1.260	0.724	2.192	0.413
	Female	52 (6.6)	331 (41.8)		33 (5.9)	251 (44.7)		0.702	0.397	1.240	0.222
Age	M ± SD	10.07 ± 4.0	9.36 ± 4.15	1.980 (0.050)	10.09 ± 4.35	9.56 ± 3.94	1.688 (0.095)	0.966	0.617	1.511	0.879
	3~6	22 (2.8)	205 (25.9)	2.688 (0.074)	19 (3.4)	126 (22.4)	0.887 (0.413)	1.426	0.698	2.913	0.329
	7~11	47 (5.9)	261 (33.0)		31 (5.5)	188 (33.5)		0.720	0.378	1.370	0.316
	12~17	39 (4.9)	217 (27.4)		28 (5.0)	170 (30.2)		0.995	0.510	1.943	0.989
Residential area	Urban	89 (11.3)	580 (73.3)	0.057 (0.812)	65 (11.6)	406 (72.2)	3.024 (0.085)	0.905	0.565	1.448	0.675
	Rural	19 (2.4)	103 (13.0)		13 (2.3)	78 (13.9)		1.359	0.470	3.925	0.570
Type of housing	General housing	28 (3.5)	141 (17.8)	1.109 (0.294)	19 (3.4)	121 (21.5)	2.744 (0.101)	1.099	0.473	2.554	0.826
	Apartment	80 (10.1)	542 (68.5)		59 (10.5)	363 (64.6)		0.913	0.454	1.531	0.730
Number in family		4.13 ± 0.75	4.18 ± 0.74	–1.046 (0.298)	4.10 ± 0.73	4.14 ± 0.75	–2.393 (0.019)	0.949	0.607	1.485	0.820
Income	Low	1 (0.1)	13 (1.6)	0.333 (0.819)	0 (0.0)	6 (1.1)	5.465 (< 0.001)				
	Lower middle	21 (2.7)	108 (13.7)		10 (1.8)	48 (8.5)		1.319	0.541	3.218	0.541
	Middle	30 (3.8)	179 (22.6)		15 (2.7)	158 (28.1)		0.460	0.204	1.033	0.060
	Upper middle	33 (4.2)	230 (29.1)		22 (3.9)	165 (29.4)		0.702	0.362	1.361	0.293
	High	23 (2.9)	153 (19.3)		31 (5.5)	107 (19.0)		2.103	0.864	5.119	0.101
Body mass index	< 5^th^ percentile	5 (0.6)	64 (8.1)	3.344 (0.021)	7 (1.3)	27 (4.9)	0.939 (0.418)	2.622	0.73	9.416	0.139
	5 – 85^th^ percentile	76 (9.6)	487 (61.6)		55 (9.9)	327 (58.8)		1.196	0.728	1.964	0.479
	85 – 95^th^ percentile	11 (1.4)	62 (8.7)		8 (1.4)	56 (10.1)		0.643	0.205	2.013	0.446
	≥ 95^th^ percentile	16 (2.0)	69 (8.7)		8 (1.4)	68 (12.2)		0.302	0.109	0.843	0.022
Subjective health status	Good	71 (9.0)	515 (65.1)	3.857 (0.023)	45 (8.0)	384 (68.3)	8.142 (< 0.001)	0.826	0.479	1.424	0.490
	Fair	31 (3.69)	151 (19.1)		27 (4.8)	87 (15.5)		1.386	0.756	2.541	0.290
	Poor	6 (0.8)	17 (2.1)		6 (1.1)	13 (2.3)		1.086	0.275	4.281	0.906
Influenza vaccination	Yes	75 (9.5)	473 (59.8)	0.001 (0.976)	56 (10.0)	381 (67.8)	4.713 (0.032)	0.830	0.513	1.341	0.444
	No	33 (4.2)	210 (26.5)		22 (3.9)	103 (18.3)		1.452	0.635	3.320	0.375
Household composition	2 generations	103 (13.0)	638 (80.7)	0.110 (0.741)	74 (13.2)	461 (82.0)	1.654 (0.201)	0.978	0.616	1.551	0.924
	3 generations or more	5 (0.6)	45 (5.7)		4 (0.7)	23 (4.1)		0.551	0.101	3.002	0.489
Parental characteristics											
Father’s smoking history	< 5 packs	1 (0.1)	8 (1.0)	1.832 (0.175)	1 (0.2)	14 (2.5)	0.621 (0.524)	0.472	0.032	7.076	0.586
	≥ 5 packs	83 (10.5)	519 (65.6)		61 (10.9)	364 (64.8)		1.029	0.613	1.727	0.913
	Never smoked	24 (3.0)	156 (19.7)		16 (2.8)	106 (18.9)		0.820	0.34	1.978	0.657
Father’s current smoking status	Daily	34 (4.3)	213 (26.9)	1.499 (0.200)	26 (4.6)	151 (26.9)	1.275 (0.284)	0.982	0.473	2.039	0.961
	Occasionally	7 (0.9)	31 (3.9)		2 (0.4)	27 (4.8)		0.154	0.024	0.999	0.05
	Former smoker	43 (5.4)	283 (35.8)		34 (6.0)	200 (35.6)		1.168	0.600	2.274	0.646
	Never smoked	24 (3.0)	156 (19.7)		16 (2.8)	106 (18.9)		0.820	0.340	1.978	0.657
Mother’s smoking history	< 5 packs	2 (0.3)	18 (2.3)	2.261 (0.124)	3 (0.5)	16 (2.8)	0.119 (0.887)	0.833	0.116	5.998	0.855
	≥ 5 packs	18 (2.3)	66 (8.3)		7 (1.2)	50 (8.9)		0.561	0.192	1.641	0.290
	Never smoked	88 (11.1)	599 (75.7)		68 (12.1)	418 (74.4)		1.071	0.649	1.768	0.787
Mother’s current smoking status	Daily	1 (0.1)	13 (1.6)	1.424 (0.235)	1 (0.2)	16 (2.8)	0.261 (0.847)	1.065	0.071	15.884	0.964
	Occasionally	2 (0.3)	14 (1.8)		0 (0.0)	6 (1.1)					
	Former smoker	17 (2.1)	57 (7.2)		9 (1.6)	44 (7.8)		0.524	0.178	1.545	0.241
	Never smoked	88 (11.1)	599 (75.7)		68 (12.1)	418 (74.4)		1.071	0.649	1.768	0.787
Mother’s age at first childbirth		27.82 (3.38)	28.56 (3.90)	–1.840 (0.068)	29.47 (3.58)	29.18 (3.76)	1.250 (0.214)	0.958	0.619	1.483	0.848
Mother’s breastfeeding history	Yes	101 (12.8)	610 (77.1)	1.467 (0.228)	74 (13.2)	430 (76.5)	0.933 (0.336)	0.998	0.634	1.571	0.994
	No	7 (0.9)	93 (9.2)		4 (0.7)	54 (9.6)		0.633	0.155	2.58	0.522
Crude								0.966	0.617	1.513	0.879
Adjusted1 (includes general characteristics)								0.929	0.596	1.446	0.743
Adjusted2 (includes general and parental characteristics)								0.93	0.603	1.434	0.742

## References

[1] Ministry of Health and Welfare. 2022 Community Integrated Health Promotion Project Guide. Ministry of Health and Welfare, Sejong.

[2] Yi Y Kim J Factors affecting asthma and atopic dermatitis in Korean children: a population-based cross-sectional survey. Child Health Nurs Res. 2015; 21: 20–27.

[3] Korea Disease Control and Prevention Agency. 2020 National Health Statistics. Available from: https://knhanes.kdca.go.kr/knhanes/sub04/sub04_04_01.do [Last accessed: 1/20/2023].

[4] Choi HG Kong IG Asthma, allergic rhinitis, and atopic dermatitis incidence in Korean adolescents before and after COVID-19. J Clin Med. 2021; 10: 3446. 34362229 10.3390/jcm10153446 PMC8347114

[5] Yang CF Yang CC Wang IJ Association between allergic diseases, allergic sensitization and attention-deficit/hyperactivity disorder in children: A large-scale, population-based study. J Chin Med Assoc. 2018; 81: 277–283. 29239851 10.1016/j.jcma.2017.07.016

[6] Im YJ Park ES Oh WO Suk MH Parenting and relationship characteristics in mothers with their children having atopic disease. J Child Health Care. 2014; 18: 215–229. 23818147 10.1177/1367493513485824

[7] Pawankar R Allergic diseases and asthma: a global public health concern and a call to action. World Allergy Organ J. 2014; 7: 12. 24940476 10.1186/1939-4551-7-12 PMC4045871

[8] Park ES Lee KH Oh WO Im Y Cho E Parenting experience of parents with chronic illness children. Child Health Nurs Res. 2015; 21: 272–284.

[9] Beacham BL Deatrick JA Children with chronic conditions: perspectives on condition management. J Pediatr Nurs. 2015; 30: 25–35. 25458105 10.1016/j.pedn.2014.10.011 PMC4291290

[10] Mendes EV Interview: The chronic conditions approach by the Unified Health System. Cien Saude Colet. 2018; 23: 431–436. 29412401 10.1590/1413-81232018232.16152017

[11] Spurr S Danford CA Roberts KJ Sheppard-LeMoine D Machado Silva-Rodrigues F Darezzo Rodrigues Nunes M Darmofal L Ersig AL Foster M Giambra B Lerret S Polfuss M Smith L Somanadhan S Fathers’ Experiences of Caring for a Child with a Chronic Illness: A Systematic Review. Children (Basel). 2023; 10: 197. 36832326 10.3390/children10020197 PMC9955404

[12] Park SE Epidemiology, virology, and clinical features of severe acute respiratory syndrome -coronavirus-2 (SARS-CoV-2; Coronavirus Disease-19). Clin Exp Pediatr. 2020; 63: 119–124. 32252141 10.3345/cep.2020.00493 PMC7170784

[13] Park J Min J Song JH Park MY Yoo H Kwon O Yang M Kim S Lee J Myong J-P The COVID-19 Pandemic Response and Its Impact on Post-Corona Health Emergency and Disaster Risk Management in Republic of Korea. Sustainability (Basel). 2023; 15: 3175.

[14] Juhn YJ Risks for infection in patients with asthma (or other atopic conditions): is asthma more than a chronic airway disease? J Allergy Clin Immunol. 2014; 134: 247–257, quiz 258-259.. 25087224 10.1016/j.jaci.2014.04.024 PMC4122981

[15] Yang JM Koh HY Moon SY Yoo IK Ha EK You S Kim SY Yon DK Lee SW Allergic disorders and susceptibility to and severity of COVID-19: A nationwide cohort study. J Allergy Clin Immunol. 2020; 146: 790–798. 32810517 10.1016/j.jaci.2020.08.008 PMC7428784

[16] Vezir E Hizal M Cura Yayla B Aykac K Yilmaz A Kaya G Oygar PD Ozsurekci Y Ceyhan M Does aeroallergen sensitivity and allergic rhinitis in children cause milder COVID-19 infection? Allergy Asthma Proc. 2021; 42: 522–529. 34871160 10.2500/aap.2021.42.210087 PMC8654388

[17] Mahdavinia M Foster KJ Jauregui E Moore D Adnan D Andy-Nweye AB Khan S Bishehsari F Asthma prolongs intubation in COVID-19. J Allergy Clin Immunol Pract. 2020; 8: 2388–2391. 32417445 10.1016/j.jaip.2020.05.006 PMC7224651

[18] Asadi-Pooya AA Simani L Central nervous system manifestations of COVID-19: A systematic review. J Neurol Sci. 2020; 413: 116832. 32299017 10.1016/j.jns.2020.116832 PMC7151535

[19] Birdi G Cooke R Knibb RC Impact of atopic dermatitis on quality of life in adults: a systematic review and meta-analysis. Int J Dermatol. 2020; 59: e75–e91. 31930494 10.1111/ijd.14763

[20] Korea National Health and Nutrition Examination Survey 2023. Korea Disease Control and Prevention Agency; [cited February 20, 2023]. Available from: https://knhanes.kdca.go.kr/knhanes/eng/index.do.

[21] Gallant AJ Nicholls LAB Rasmussen S Cogan N Young D Williams L Changes in attitudes to vaccination as a result of the COVID-19 pandemic: A longitudinal study of older adults in the UK. PLoS One. 2021; 16: e0261844. 34941951 10.1371/journal.pone.0261844 PMC8699689

[22] Ullah MA Moin AT Araf Y Bhuiyan AR Griffiths MD Gozal D Potential effects of the COVID-19 pandemic on future birth rate. Front Public Health. 2020; 8: 578438. 33363080 10.3389/fpubh.2020.578438 PMC7758229

[23] Beasley R Semprini A Mitchell EA Risk factors for asthma: is prevention possible? Lancet. 2015; 386: 1075–1085. 26382999 10.1016/S0140-6736(15)00156-7

[24] Patel S Henderson J Jeffreys M Associations between socioeconomic position and asthma: findings from a historical cohort. Eur J Epidemiol. 2017; 32: 797–805. 22696048 10.1007/s10654-012-9703-9 PMC3444704

[25] Belgrave DC Granell R Simpson A Guiver J Bishop C Buchan I Henderson AJ Custovic A Developmental profiles of eczema, wheeze, and rhinitis: two population-based birth cohort studies. PLoS Med. 2014; 11: e1001748. 25335105 10.1371/journal.pmed.1001748 PMC4204810

[26] Lee S Wang HY Kim E Hwang HJ Choi E Lee H Choi EH Clinical characteristics and genetic variation in atopic dermatitis patients with and without allergic contact dermatitis. Eur J Dermatol. 2018; 28: 637–643. 30530433 10.1684/ejd.2018.3422

[27] Bantz SK Zhu Z Zheng T The atopic march: Progression from atopic dermatitis to allergic rhinitis and asthma. J Clin Cell Immunol. 2014; 5: 202. 25419479 10.4172/2155-9899.1000202 PMC4240310

[28] Paller AS Spergel JM Mina-Osorio P Irvine AD The atopic march and atopic multimorbidity: Many trajectories, many pathways. J Allergy Clin Immunol. 2019; 143: 46–55. 30458183 10.1016/j.jaci.2018.11.006

[29] Lee JY Kim J Ahn K Time trends in the prevalence of atopic dermatitis in Korean children according to age. Allergy Asthma Immunol Res. 2022; 14: 123–130. 34983112 10.4168/aair.2022.14.1.123 PMC8724825

[30] Wollenberg A Werfel T Ring J Ott H Gieler U Weidinger S Atopic dermatitis in children and adults: Diagnosis and treatment. Dtsch Arztebl Int. 2023; 120: 224–234. 36747484 10.3238/arztebl.m2023.0011 PMC10277810

[31] Guo Y Zhang KY Zou YF Yu B National situation, trends, and predictions of disease burden of atopic dermatitis in Chinese children and adolescents. Front Microbiol. 2023; 14: 1161969. 37396371 10.3389/fmicb.2023.1161969 PMC10308015

[32] Silverberg JI Hanifin JM Simpson EL Climatic factors are associated with childhood eczema prevalence in the United States. J Invest Dermatol. 2015; 135: 1905–1907. 23334343 10.1038/jid.2013.19 PMC3646081

[33] Bylund S Kobyletzki LB Svalstedt M Svensson Å Prevalence and incidence of atopic dermatitis: a systematic review. Acta Derm Venereol. 2020; 100: adv00160. 32412646 10.2340/00015555-3510 PMC9189744

[34] Flohr C Mann J New insights into the epidemiology of childhood atopic dermatitis. Allergy. 2014; 69: 3–16. 24417229 10.1111/all.12270

[35] Pelucchi C Galeone C Bach JF La Vecchia C Chatenoud L Pet exposure and risk of atopic dermatitis at the pediatric age: a meta-analysis of birth cohort studies. J Allergy Clin Immunol. 2013; 132: 616–622. 23711545 10.1016/j.jaci.2013.04.009

[36] La Verde M Torella M Riemma G Narciso G Iavarone I Gliubizzi L Palma M Morlando M Colacurci N De Franciscis P Incidence of gestational diabetes mellitus before and after the Covid-19 lockdown: A retrospective cohort study. J Obstet Gynaecol Res. 2022; 48: 1126–1131. 35199420 10.1111/jog.15205 PMC9115303

[37] Bousquet J Anto JM Iaccarino G Is diet partly responsible for differences in COVID-19 death rates between and within countries? Clin Transl Allergy. 2021; 11: 1–11. 10.1186/s13601-020-00323-0 PMC7250534 32499909

[38] Li X Li T Chen N Wang X Yang J Bi L Influence of COVID-19 pandemic on inhaled allergens in children with allergic diseases, Henan, China. J Med Virol. 2023; 95: e28409. 36519585 10.1002/jmv.28409 PMC9877546

